# Conditions for CaCO_3_ Biomineralization by *Trichoderma Reesei* with the Perspective of Developing Fungi‐Mediated Self‐Healing Concrete

**DOI:** 10.1002/gch2.202300160

**Published:** 2023-12-21

**Authors:** Aurélie Van Wylick, Hubert Rahier, Lars De Laet, Eveline Peeters

**Affiliations:** ^1^ Research Group of Microbiology Department of Bioengineering Sciences Vrije Universiteit Brussel Pleinlaan 2 Brussels B‐1050 Belgium; ^2^ Research Group of Physical Chemistry and Polymer Science Department of Materials and Chemistry Vrije Universiteit Brussel Pleinlaan 2 Brussels B‐1050 Belgium; ^3^ Research Group of Architectural Engineering Department of Architectural Engineering Vrije Universiteit Brussel Pleinlaan 2 Brussels B‐1050 Belgium

**Keywords:** biomineralization, calcium carbonate, fungi, self‐healing concrete, trichoderma reesei

## Abstract

Concrete, a widely used building material, often suffers from cracks that lead to corrosion and degradation. A promising solution to enhance its durability is the use of fungi as self‐healing agents, specifically by harnessing their ability to promote calcium carbonate (CaCO_3_) precipitation on their cell walls. However, the ideal conditions for CaCO_3_ precipitation by the filamentous fungal species *Trichoderma reesei* are still unclear. In this study, the biomineralization properties of *T. reesei* in liquid media are investigated. Two different calcium sources, calcium chloride (CaCl_2_) and calcium lactate are tested, at varying concentrations and in the presence of different nutritional sources that support growth of *T. reesei*. This study also explores the effects on fungal growth upon adding cement to the medium. Calcium lactate promotes greater fungal biomass production, although less crystals are formed as compared to samples with CaCl_2_. An increasing calcium concentration positively influences fungal growth and precipitation, but this effect is hindered upon the addition of cement. The highest amounts of biomass and calcium carbonate precipitation are achieved with potato dextrose broth as a nutritional source. By identifying the optimal conditions for CaCO_3_ precipitation by *T. reesei*, this study highlights its potential as a self‐healing agent in concrete.

## Graphical abstract

Biomineralization properties of *Trichoderma reesei* are investigated for calcium carbonate precipitation in concrete‐relevant conditions. Optimal conditions are determined, with calcium lactate promoting greater fungal biomass production compared to calcium chloride, albeit with fewer crystals. An increased calcium concentration enhances fungal growth and precipitation, while cement addition hinders this effect. The highest biomass and CaCO_3_ precipitation occur in a potato dextrose broth medium.

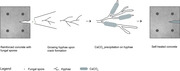

## 1 Introduction

Given the increasing urge to find durable solutions in the construction sector, concrete has become a major concern on a global scale. Being a vital element in our built environment, as it is used in 80% of the construction cases,^[^ ^1^, ^2^ ^]^ concrete is susceptible to crack formation owing to its hardening shrinkage, low tensile strength and brittle behaviour. These cracks form pathways for water, oxygen and CO_2_ causing freeze‐thaw damage, chemical attack, reinforcement corrosion and internal expansion over time.^[^ ^3^, ^4^ ^]^ With a global cost of 3 billion euros in 2022 for the concrete repair mortars market alone,^[^ ^5^ ^]^ endless inspection, maintenance and renovation are a plague to the economy. Additionally, cement production accounts for 8% of the global anthropogenic CO_2_ emissions caused by the calcination of limestone and fuel combustion.^[^ ^1^ ^]^ With a production of >4 billion tonnes of cement yearly,^[^ ^6^ ^]^ the environmental alarm has been going on with increasing sound.

To address the critical challenges linked to concrete that impact both the economy and environment, the concept of self‐healing concrete has been proposed as a solution. Through autogenous self‐healing, the material possesses inherent capabilities to repair small cracks up to 0.2 mm in width through two primary mechanisms: carbonation and further hydration of unhydrated cementitious particles near the cracks.^[^ ^7^, ^8^ ^]^ Carbonation involves the reaction between atmospheric CO_2_ and portlandite (Ca(OH)_2_), resulting in the formation of calcium carbonate (CaCO_3_). The autogenous presence of CaCO_3_ ensures compatibility and bonding with concrete, making it a highly desirable self‐healing product. To encourage autogenous self‐healing, the addition of superabsorbent polymers (SAPs) has been investigated as well. These polymers can absorb large amounts of water and their consequent swelling capacity allows them to (partially) block cracks.^[^ ^3^, ^8^, ^9^, ^10^, ^11^ ^]^ Additionally, they can release this water in drier periods, thereby promoting the concrete's autogenous self‐healing mechanisms.^[^ ^9^, ^10^, ^11^ ^]^ The crack size that can be healed is however limited as well to cracks <0.2 mm.^[^ ^8^ ^]^ Autonomous self‐healing methods require a self‐healing agent, such as specific bacteria and fungi capable of CaCO_3_‐producing biomineralization.^[^ ^4^, ^12^, ^13^, ^14^, ^15^, ^16^, ^17^, ^18^, ^19^ ^]^ These approaches rely on the addition of microbial cells and nutrients to the concrete mixture during the manufacturing process.

Despite the promise of microbe‐mediated self‐healing concrete applications, it should be considered that concrete presents a challenging and unfavourable environment for microorganisms due to its alkaline characteristics and the limited availability of nutrients, oxygen and water. To ensure the survival of microorganisms under such harsh conditions, microbial spores are utilized due to their metabolic inactivity, high resistance and prolonged viability.^[^ ^4^, ^14^, ^20^ ^]^ Activation of these spores occurs when cracks form and allow the entry of water and oxygen, triggering spore germination and subsequent vegetative growth. This growth then enables the biomineralization of CaCO_3_ on the surfaces of microbial cell walls, leading to the healing of cracks.^[^ ^4^, ^14^, ^16^, ^20^ ^]^ The crystal formation is a result of the reaction between Ca^2+^ ions present on the cell wall with CO_3_^2−^ ions.^[^ ^20^ ^]^ Microbially induced calcite precipitation (MICP) has been extensively researched for bacteria.^[^ ^13^, ^15^, ^16^, ^17^, ^21^ ^]^ In this case, bacteria‐mediated self‐healing concrete has been demonstrated to achieve crack healing of cracks up to 1 mm.^[^ ^12^ ^]^ The combination of SAPs and bacteria has been studied as well. SAPs aid the germination of the spores and bacterial activity by supplying water.^[^ ^8^ ^]^ Additionally, the ability of SAPs to swell and block the crack is maintained, thus improving autogenous crack healing by providing water for further hydration and CaCO_3_ precipitation.^[^ ^8^ ^]^ Cracks with a width up to 0.7 mm can be healed by 70%.^[^ ^8^ ^]^

Increasing attention has been directed toward the use of fungi given their extensive network‐like growth, thereby offering more sites for CaCO_3_ precipitation.^[^ ^4^, ^14^, ^19^, ^22^ ^]^ But also in this case, the alkaline environment of concrete poses challenges to fungal survival and growth. To select suitable fungal species or strains as self‐healing agents in concrete, stringent criteria must be met such as non‐pathogenicity, capability of producing highly resistant spores, ability for vegetative growth in crack‐forming conditions, survival and growth in harsh alkaline environments and promotion of CaCO_3_ precipitation at the cell wall surface.^[^ ^20^ ^]^ Several well‐characterized model species, such as *Aspergillus nidulans*, *Trichoderma reesei*, *Fusarium oxysporum*, *Neurospora crassa, Rhizopus oryzae*, and *Trichoderma longibrachiatum* have been identified as potentially capable of growing and precipitating CaCO_3_ in concrete‐relevant environments.^[^ ^4^, ^14^, ^19^, ^22^, ^23^, ^24^ ^]^ Additionally, novel isolates sampled from relevant natural or anthropogenic habitats, for example from a limestone cave, were characterized for their growth and CaCO_3_ precipitation abilities in concrete‐like conditions.^[^ ^25^ ^]^ This study demonstrated that the isolated strain *Trichoderma sp*. SVS008 was one the strains with the highest potential for the application.^[^ ^25^ ^]^

*Trichoderma sp*. belongs to the phylum Ascomycota within the class Sordariomycetes in the order of the Hypocreales.^[^ ^26^ ^]^ The taxon *Trichoderma* has many ubiquitously distributed species, these commonly thrive in natural soils, root ecosystems and decaying wood.^[^ ^27^, ^28^ ^]^ *Trichoderma* species are of great commercial interest because of their efficient production of many extracellular enzymes^[^ ^29^ ^]^ and their ability to be used as biofertilizers and biofungicides.^[^ ^26^ ^]^ *Trichoderma reesei* specifically is the model organism for industrial production of cellulolytic enzymes which is of importance to the biotechnology industry, for example to produce biofuels.^[^ ^30^ ^]^ As a well‐characterized species, pioneering studies have included *T. reesei* in their screening to uncover potential candidates for the application of self‐healing concrete with fungi.^[^ ^4^, ^14^ ^]^ This led to the conclusion that the spores of *T. reesei* could germinate and grow in a mycelial network on a layer of potato dextrose agar (PDA) that was poured on a 7‐day cured concrete.^[^ ^14^ ^]^ The samples were analysed with X‐ray diffraction (XRD), scanning electron microscopy (SEM) and energy dispersive X‐ray spectroscopy (EDX), these techniques are used to respectively characterize crystalline materials,^[^ ^31^ ^]^ visualize the morphology of fungal precipitates and characterize the composition of the precipitates.^[^ ^14^ ^]^ Results confirmed the presence of CaCO_3_ on the hyphae.

This study aims to further investigate the potential of *T. reesei* for the development of self‐healing concrete by determining optimal conditions for CaCO_3_ precipitation. The experimental design is informed by a comprehensive literature review on bacteria‐based self‐healing concrete,^[^ ^13^, ^15^, ^16^, ^17^, ^20^, ^21^, ^32^ ^]^ recent advancements in fungi‐mediated self‐healing concrete^[^ ^4^, ^14^, ^19^, ^33^ ^]^ and studies on CaCO_3_ biomineralization by fungi.^[^ ^18^, ^33^ ^]^ The biomineralization process of CaCO_3_ by *T. reesei* is examined in liquid media containing various nutrients, urea and different calcium sources and concentrations. The chosen approach in this study focuses on the ureolytic pathway, which involves the breakdown of urea to generate carbonate ions (CO_3_^2−^).^[^ ^18^, ^33^ ^]^ Furthermore, the impact of adding cement to the media is explored to assess fungal growth under concrete‐like conditions. Considering the alkaline pH of concrete, the potential use of a pH buffer in the media is also investigated, with the aim of mitigating adverse effects of pH on the survival of *T. reesei*. An overview of the experimental design in this study is shown in Figure S1 (Supporting Information).

## 2 Results and Discussion

### 2.1 Effects of Calcium Sources on Fungal Growth and CaCO_3_ Precipitation

**Figure 1:**
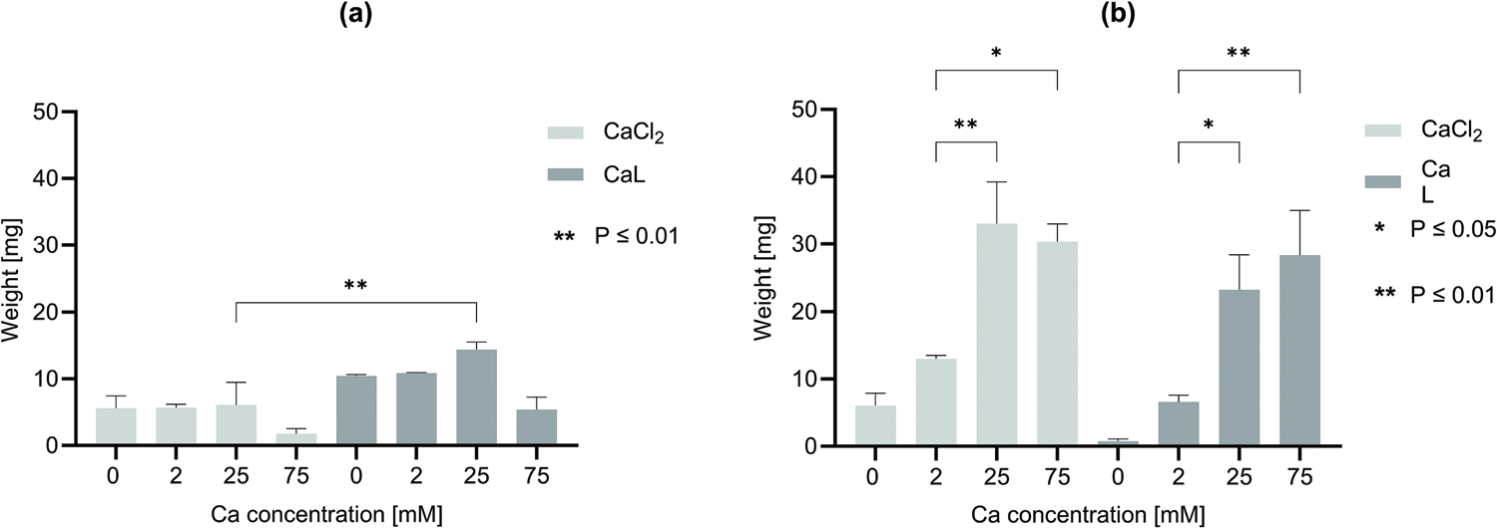
Biomass and CaCO_3_ formation upon growing *T. reesei* in the presence of exogenous calcium sources. Graphical presentation of a) the dry weight of the grown biomass and b) precipitated CaCO_3_ at the end of the experiment. Next to the calcium source, all media contained 1.5% corn steep liquor (CSL) and 2% urea. The statistical relevance was determined by performing a One‐way ANOVA in GraphPad, Prism.

In the first section of the study, we set out to investigate the effects of adding different calcium sources on growth and CaCO_3_ precipitation in liquid cultures. This was done by performing a growth experiment in the presence of either CaCl_2_ or calcium lactate (CaL) at different concentrations and measuring both biomass and CaCO_3_ weight (**Figure** **1**; Table S1, Supporting Information). Both calcium sources have been investigated for bacteria‐based self‐healing concrete,^[^ ^2^, ^34^, ^35^, ^36^, ^37^, ^38^ ^]^ however for fungi a comparison has not yet been made. Another calcium source of interest is for example calcium acetate.^[^ ^37^, ^38^ ^]^ Growth was clearly stimulated by the presence of an exogenous calcium source, up to a value of 75 mM. In the presence of CaCl_2_ the formed biomass remained constant at 2 and 25 mM as compared to the negative control but decreased significantly to 1.8 mg at 75 mM. In contrast, CaL resulted in (almost) double the mean biomass at 0, 2, and 25 mM compared to CaCl_2_, reaching ≈11 mg (0 and 2 mM) and 14 mg (25 mM). In both cases, a calcium concentration of 75 mM resulted in a decrease in formed biomass.

In terms of crystal precipitation, the samples with CaCl_2_ demonstrated higher amounts of precipitated CaCO_3_ compared to the corresponding calcium concentrations in the CaL samples (Figure 1; Table S1, Supporting Information). Generally, precipitation increased with higher calcium concentrations. Comparing both calcium sources, the addition of CaCl_2_ yielded the highest absolute amounts of CaCO_3_, reaching 33.0 and 30.4 mg for CaCl_2_ concentrations of 25 and 75 mM, respectively. In conclusion, the choice of calcium source impacts both crystal formation and fungal growth, with CaCl_2_ promoting crystal formation and CaL stimulating fungal growth.

The pH values for the respective media and concentrations were similar and followed the same trendline (Table S1, Supporting Information).

### 2.2 Impact of the Presence of Cement on Growth and Precipitation

**Figure 2:**
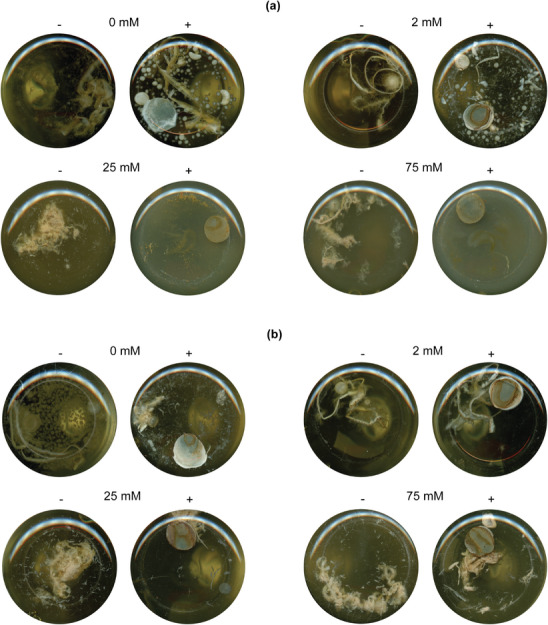
Grown fungal biomass for cultures containing a) CaCl_2_ and b) CaL in the absence (−) and presence (+) of a cement paste piece. These pictures are taken from the bottom of the flask of the liquid culture. For each concentration of calcium source, the reference sample is shown on the left (−) and the sample with a piece of cement paste on the right (+). Each picture is a representative result for replicate experiments.

In order to simulate the actual concrete environment, liquid cultures were then incubated in the presence of a cement paste piece (**Figure** **2**). These experiments were not subjected to quantitative analysis but rather served as exploratory observations. Growth was clearly inhibited in cultures containing cement and CaCl_2_ at concentrations of 25 and 75 mM. Conversely, growth was still observed in samples containing the same concentrations of CaL with cement. It is important to note that this growth was not consistently observed in all replicates. Overall, these results suggest that the combination of a high calcium concentration and the presence of cement may have an adverse effect on fungal growth. This should be considered during the design of further experimental set‐ups for the development of self‐healing concrete with *T. reesei*.

Based on all findings thus far, 25 mM CaL was chosen as a preferred condition for the next part of the study. This calcium source and concentration leads to an increased fungal biomass and potential for CaCO_3_ precipitation as compared to CaCl_2_. Moreover, the presence of chloride ions could be detrimental for the steel reinforcement.^[^ ^16^ ^]^ Although growth was observed in the presence of cement for some replicates, it should be taken into account that this was not a consistent result.

### 2.3 Influence of Nutrient Media on Fungal Growth and CaCO_3_ Precipitation

**Figure 3:**
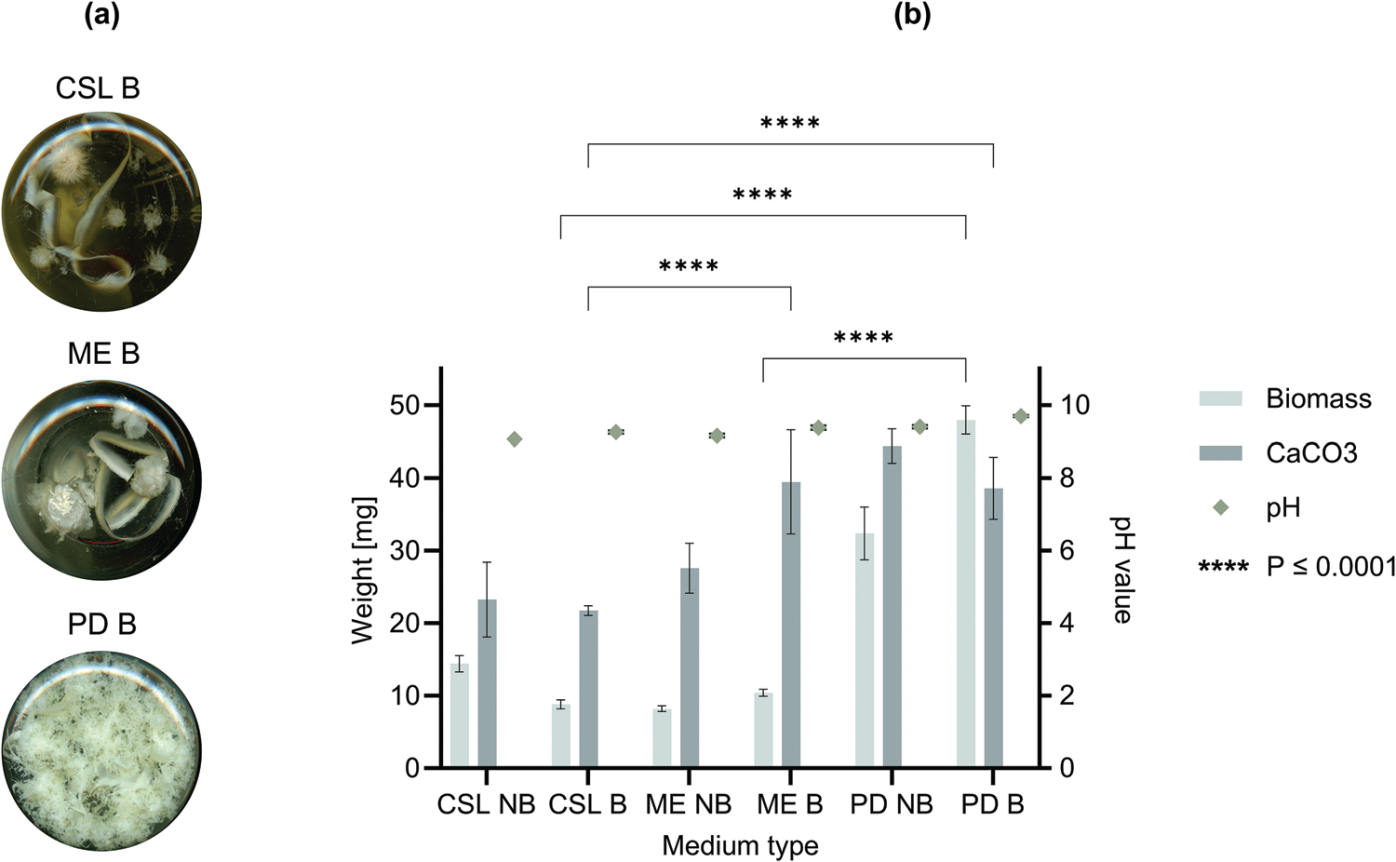
Biomass and CaCO_3_ formation upon growing *T. reesei* in presence of different media, without or with a pH buffer. a) Grown fungal biomass for cultures containing buffered media, namely corn steep liquor (CSL), malt extract (ME) or potato dextrose broth (PD) medium. These pictures are taken from the bottom of the flask of the liquid culture. Each picture is a representative result for replicate experiments. b) Biomass and CaCO_3_ quantification. Graphical presentation of the dry weight of the grown biomass, precipitated CaCO_3_ and pH value at the end of the experiment for samples grown in non‐buffered (NB) and buffered (B) media containing CSL, ME or PD as nutrient source. All media contained 25 mm CaL and 2% urea. The statistical relevance is shown for the buffered media and was determined by performing a Two‐way ANOVA in GraphPad, Prism.

To explore the impact of medium composition on growth and CaCO_3_ precipitation, we next conducted experiments comparing different nutritional conditions, moving away from the corn steep liquor (CSL) medium consistently used in previous experiments (**Figure** **3**; Table S2, Supporting Information). CSL was initially selected as a nutrient source because it has been investigated for bacteria as well.^[^ ^32^ ^]^ In addition, we set out to evaluate the impact of a pH buffer on growth and precipitation. With regards to potential applications, the use of a buffered medium could assist in maintaining a stable pH value, thereby reducing stress when *T. reesei* is exposed to the alkaline environment of concrete.

The results revealed distinct preferences of *T. reesei* for different nutrient media in terms of fungal growth and CaCO_3_ precipitation (Figure 3; Table S2, Supporting Information). Among the tested media, potato dextrose broth (PD) supported best growth of *T. reesei*, as evidenced by the abundant presence of biomass in the culture at the end of the experiment (Figure 3a). This is corroborated by the quantitative data obtained for the cultures in PD, with an average biomass dry weight of 32.4 and 48.1 mg for samples without (NB) and with a buffer (B), respectively (Figure 3b; Table S2, Supporting Information). Potato dextrose is the main nutritional source used for the cultivation of *T. reesei* ^[^ ^39^, ^40^, ^41^, ^42^ ^]^ and has been used previously to assess growth on concrete plates.^[^ ^14^ ^]^ PD was therefore included in this study. The PD medium without a buffer yielded the highest amount of precipitation, measuring 44.4 mg, followed by the buffered PD medium with 38.6 mg, which was very similar to the buffered malt extract (ME) medium with 38.5 mg. Interestingly, the addition of a buffer positively influenced both fungal growth and CaCO_3_ precipitation in the ME media and fungal growth in the PD media, whereas an opposite effect was observed in the CSL medium. Moreover, the pH was consistently higher in the presence of a buffer at the end of the experiments (Figure 3b).

### 2.4 Visualization of Hyphal Structures and CaCO_3_ Crystals

**Figure 4:**
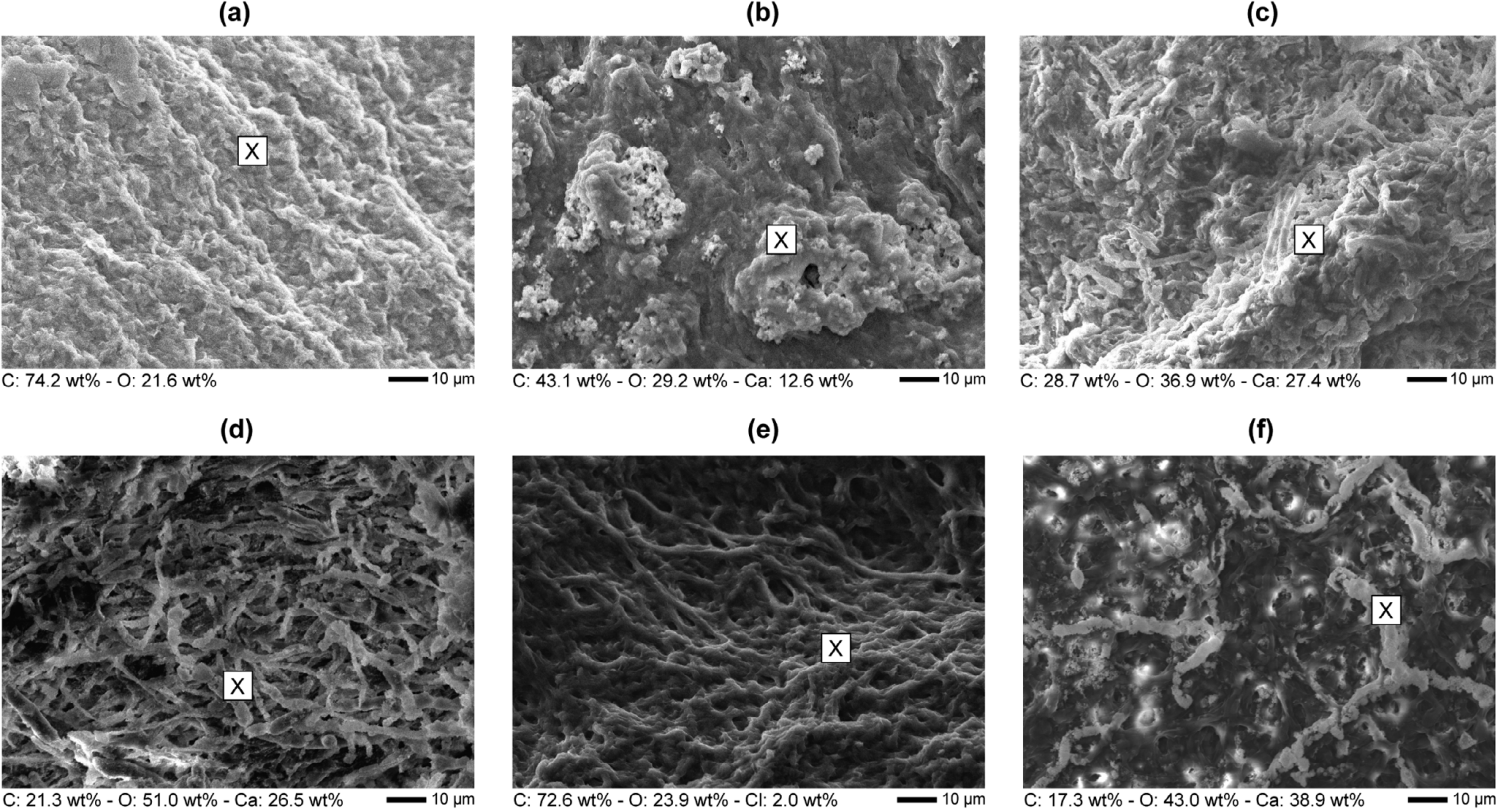
Visualization of hyphal structures and CaCO_3_ crystals by SEM‐EDX. Representative SEM images of samples grown in CSL medium with a) 0 mm CaCl_2_, b) 0 mm CaCl_2_ CEM and c) 25 mm CaCl_2_. Representative SEM images of samples grown in (d) ME B medium, e) ME B medium after HCl treatment and f) PD B medium. B indicates the presence of a pH buffer. The weight percentages of the most abundant and relevant elements in the samples are given. The location of the spectrum is indicated by a cross on the SEM image. The abbreviation “CEM” refers to the presence of a cement paste piece in the growth medium. All media contain 2% urea.

Next, Scanning Electron Microscopy (SEM) with Energy Dispersive X‐ray Diffraction (EDX) was employed to examine the morphology of fungal hyphae and, if present, the CaCO_3_ crystals (**Figure** **4**). EDX enabled to confirm the chemical composition of the crystals. In the initial experiment, the analysis was performed on samples from cultures with CSL medium and different CaCl_2_ concentrations containing a piece of cement paste (CEM) (Figure 4a–c). The results for the negative control without addition of an exogenous calcium source (Figure 4a) and the corresponding CEM sample (Figure 4b) demonstrated the occurrence of CaCO_3_ precipitation when the cement acted as the sole calcium source. While the negative control without CaCl_2_ and a piece of cement paste primarily exhibited organic material, the CEM sample exhibited evident crystals on the hyphae. In the sample from a culture with 25 mm CaCl_2_, the hyphae were completely enveloped by the precipitation (Figure 4c).

In a second experiment, SEM‐EDX images were generated for samples of fungal biomass grown in ME and PD medium in the presence of CaL, as well as the fungal biomass after HCl treatment, which was done for CaCO_3_ quantification (Figure 4d–f). These results demonstrated the efficacy of the HCl treatment. While CaCO_3_ was clearly observed for the samples before HCl treatment (Figure 4d), it was absent in the sample following HCl treatment (Figure 4e). Here, the hyphae were no longer encrusted by the crystals underscoring their dissolution into CaCl_2_, H_2_O and CO_2_. Indeed, the release of CO_2_ was observed during the addition of the HCl solution, by the formation of small bubbles. EDX analysis confirmed the presence of Cl in the sample.

### 2.5 Chemical Analysis of CaCO_3_ Precipitation on Fungal Hyphae

**Figure 5:**
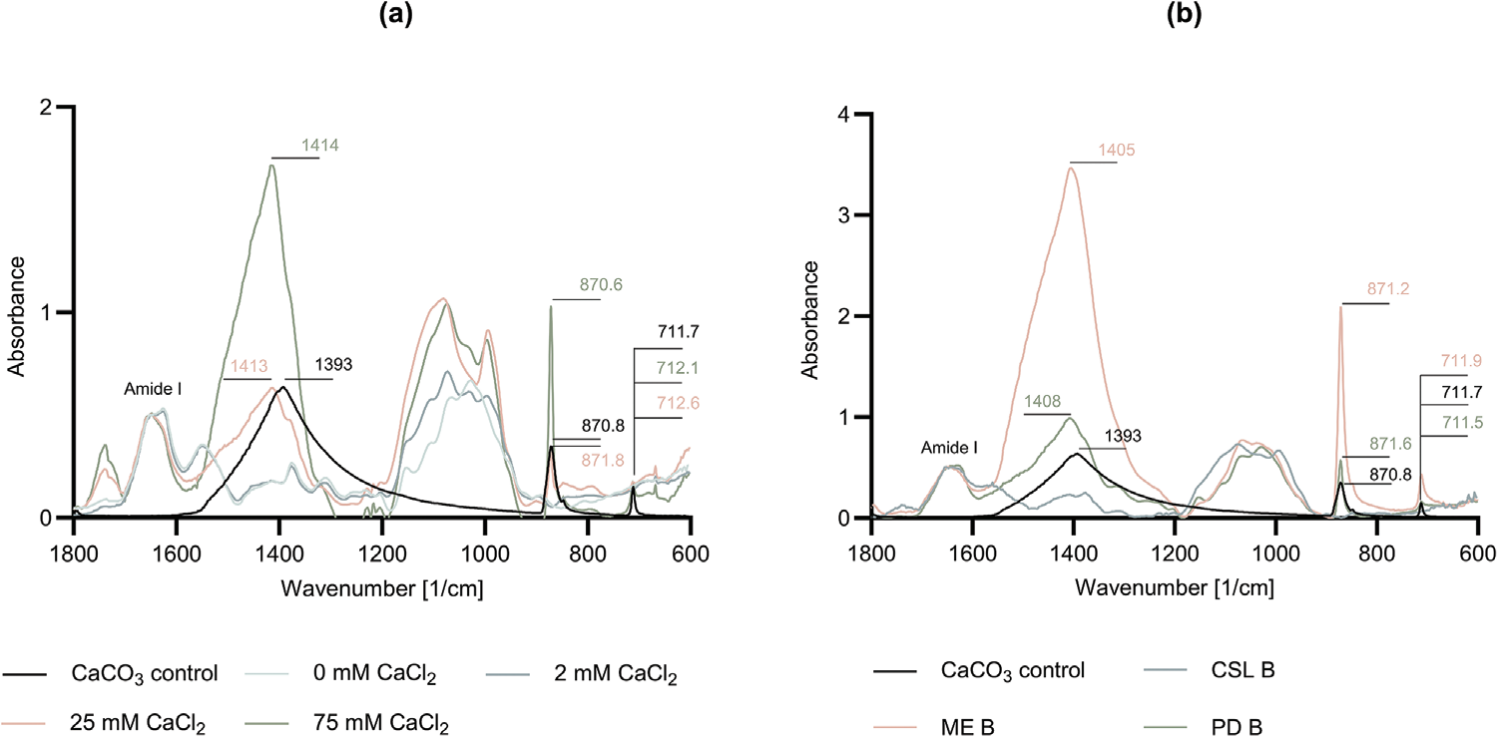
Chemical analysis of CaCO_3_ precipitation on fungal hyphae with FTIR. Absorbance spectra are shown for samples from cultures cultivated with (a) CaCl_2_ and (b) in different media. The wavenumbers of the relevant peaks are indicated, as well as the Amide I region. It should be noted that the y‐axis of the graphs were scaled differently to ensure accurate visualization in each experiment.

Finally, Fourier Transform Infrared Spectroscopy (FTIR) was employed to further analyze the chemical composition of the samples in cultures with different media and the addition of either CaCl_2_ or CaL (**Figure** **5**; Figure S2 and Tables S3 and S4, Supporting Information).

For the samples derived from cultures grown in CSL in the presence of CaCl_2_, FTIR confirmed the presence of CaCO_3_ on the fungal hyphae for samples with a CaCl_2_ concentration of 25 and 75 mM (Figure 5a; Table S3, Supporting Information). Similarly, in the case of samples with CaL, growth in the presence of 75 mm CaL indicated CaCO_3_ formation (Figure S2, Supporting Information). Factors such as crystal location, measurement frequency, and experimental variables likely contributed to the lack of observing CaCO_3_ at lower CaL concentrations. Experiments conducted with CSL, ME and PD mediums consistently resulted in FTIR spectra that exhibited relevant peaks (Figure 5b; Table S4, Supporting Information). Notably, both ME B and PD B samples displayed peaks resembling those of the control CaCO_3_ sample. Therefore, we can conclude that it is confirmed that all medium compositions can support the capability of *T. reesei* to precipitate CaCO_3_, as required for the self‐healing concrete application.

## 3 Conclusion

In this work, we have shown that the choice of calcium source and concentration exerts a significant influence on fungal growth of *T. reesei* and CaCO_3_ precipitation. Calcium lactate demonstrates a notable increase in biomass dry weight compared to calcium chloride, with the weight of precipitated crystals increasing with increasing calcium concentrations. The final pH values for calcium lactate and calcium chloride samples follow a similar trendline with decreasing values for increasing calcium concentrations. Among the nutritional sources, potato dextrose broth demonstrates to be the best medium with the highest fungal biomass and CaCO_3_ precipitation yields. Based on these findings, an optimal biomineralization medium for *T. reesei* can be proposed, consisting of 1.5% potato dextrose broth, 25 mm calcium lactate, 2% urea, and a starting pH value of 5 achieved through the addition of a citrate buffer. Future research endeavours may focus on exploring higher pH levels and modifying calcium concentrations to further investigate these conditions.

This research is a fundamental step toward the implementation of fungal spores into the concrete mix. The final goal is to encapsulate the spores to protect the organism from the mixing, hardening and harsh environment of concrete. The capsules aim to ensure the spores’ survivability and provide the necessary nutrients and additives to promote fungal growth and CaCO_3_ precipitation. The study demonstrates the ability of *T. reesei* to precipitate CaCO_3_ crystals when growing in suitable liquid conditions and proposes an optimal biomineralization medium that could be used in further explorations on the encapsulation of the spores. Biomineralization in liquid media has already been demonstrated for other species as well, such as *Neurospora crassa* ^[^ ^18^, ^33^ ^]^ and *Fusarium oxysporum*,^[^ ^19^ ^]^ these studies focused on the growth and precipitation on a surface of respectively mortar and concrete and yielded positive results. Although the addition of cement was considered in this research as well, it was rather explorative and requires a more in‐depth investigation.

## 4 Experimental Section

### Microbial Strains and Growth Conditions

The fungal strain *Trichoderma reesei* DSM 768 (Leibniz Institute DSMZ‐German Collection of Microorganisms and Cell Cultures, ATCC13631) was cultivated on malt extract agar (MEA) in 90 mm diameter Petri dishes under 30°C with 12 h light/dark cycles. After 5 days of incubation, cylindrical disks were prepared from the 5‐day‐old cultures using a 200 µl pipette tip. These disks were then used to prepare liquid cultures by inoculating 100 mL volume Erlenmeyer flasks containing the different media. The experimental design of the entire study is depicted in a schematical workflow diagram in Figure S1 (Supporting Information).

In the first set of experiments, *T. reesei* was cultivated in corn steep liquor (CSL) medium, consisting of 1.5% CSL and 2% urea, but with a different calcium source, which was either CaCl_2_ or CaL at three different concentrations (2, 25 and 75 mM). A negative control without an exogenous calcium source was included as well. The pH of the media was measured and adjusted to 5 before autoclaving. pH measurements were taken at the start and end of each experiment. In half of the cultures, circular cement paste pieces, cured for 1 week, were added in a similar manner as described.^[^ ^43^ ^]^ The cement (CEM III/B 42.5 N‐LH/SR LA) was obtained from Holcim,^[^ ^44^ ^]^ with the cement paste pieces having been prepared with a water‐to‐cement ratio (w/c) of 0.45, weighing ≈0.85 g each. The cultures containing cement were solely utilized for qualitative analyses and to this end, pictures were taken of the culture flasks.

In a second set of experiments, six different types of media were inoculated and compared to assess the influence of the nutrient (CSL, malt extract (ME) and potato dextrose broth (PD)) and the absence or presence of a pH buffer (0.1 m citrate buffer) on fungal growth and CaCO_3_ precipitation. The pH of all media was adjusted to 5 before autoclaving at 121°C for 15 min before inoculation. Urea was added after filtration‐sterilization. Cultures were incubated under shaking conditions at 120 rpm and 30 °C for 1 week. All experiments were conducted in at least triplicates. pH measurements were taken at the beginning and end of each experiment.

### Biomass Weighing

Biomass and cement pieces were separated from the media with a vacuum pump and filter paper and then placed in 90 mm Petri dishes. The biomass was denatured by adding 10 mL of 70% ethanol to the plates, after which the plates were placed in the oven and dried at 40 °C for 24 h. The dried and killed biomass was finally weighed with an analytical balance.

### Quantification of CaCO_3_ Precipitation

Precipitation was quantified by immersing the dried biomass in a 2 m HCl solution for 15 min. Samples were then rinsed with distilled water and the resulting biomass was retrieved using a vacuum pump. After drying in an oven at 40 °C for 48 h, the samples were weighed again and the amount of CaCO_3_ crystals was determined.

### Scanning Electron Microscopy with Energy Dispersive X‐Ray Spectroscopy

SEM combined with EDX was performed using a JSM‐IT300 InTouchScope as a means to visualize the morphology of fungal CaCO_3_ precipitates and to characterize the composition of the crystals. The dried biomass was first crushed into a powder and then sputter coated with a 1.3 nm layer of gold. The samples were mounted on the sample carriers with carbon tape. For both the SEM and EDX analyses, an accelerating voltage of 15 kV was used.

### Fourier Transform Infrared Spectroscopy

FTIR was employed to confirm the presence of CaCO_3_ by comparing the spectra to that obtained with a control sample of pure CaCO_3_. The spectra were obtained using a Nicolet 6700 FT‐IR spectrometer from Thermo Fisher Scientific. Prior to analysis, the spectra were scaled using the Amide I peak, which arises from protein‐induced C═O stretching vibrations at wavenumbers around ≈1630–1645 cm^−1^,^[^ ^45^ ^]^ ≈1635 cm^−1[^ ^46^ ^]^ and ≈1650 cm^−1[^ ^47^, ^48^ ^]^ and then multiplied by a factor of 0.5. All spectra represented the mean values for each type of sample. The x‐axis of the spectra was limited to a wavenumber of 1800 cm^−1^ to enhance the visibility of the relevant peaks.

### Statistical Analysis

The statistical relevance was determined by performing either a One‐way or a Two‐way ANOVA in GraphPad, Prism. Standard deviations and p‐values are given in the corresponding figures.

## Conflict of Interest

The authors declare no conflict of interest.

## Supporting information

Supporting Information

## Data Availability

The data that support the findings of this study are available in the supplementary material of this article.
