# Supplementary material for: Conditions for CaCO3 Biomineralization by Trichoderma Reesei with the Perspective of Developing Fungi‐Mediated Self‐Healing Concrete
Source: Glob Chall. 2023 Dec 21;8(1):2300160. doi: 10.1002/gch2.202300160 (PMC10784186; doi:10.1002/gch2.202300160)
Supplement: Supplementary file 1 — Supporting Information [file GCH2-8-2300160-s001.pdf]

Global Challenges
Open Access

Supporting Information

for *Global Challenges.*, DOI 10.1002/gch2.202300160

Conditions for $CaCO_3$ Biomineralization by *Trichoderma Reesei* with the Perspective of Developing Fungi-Mediated Self-Healing Concrete

*Aurélie Van Wylick, Hubert Rahier, Lars De Laet and Eveline Peeters**

Supporting Information

Conditions for $CaCO_3$ Biomineralization by *Trichoderma reesei* with the Perspective of Developing Fungi-Mediated Self-Healing Concrete

Aurélie Van Wylick, Hubert Rahier, Lars De Laet, and Eveline Peeters,*

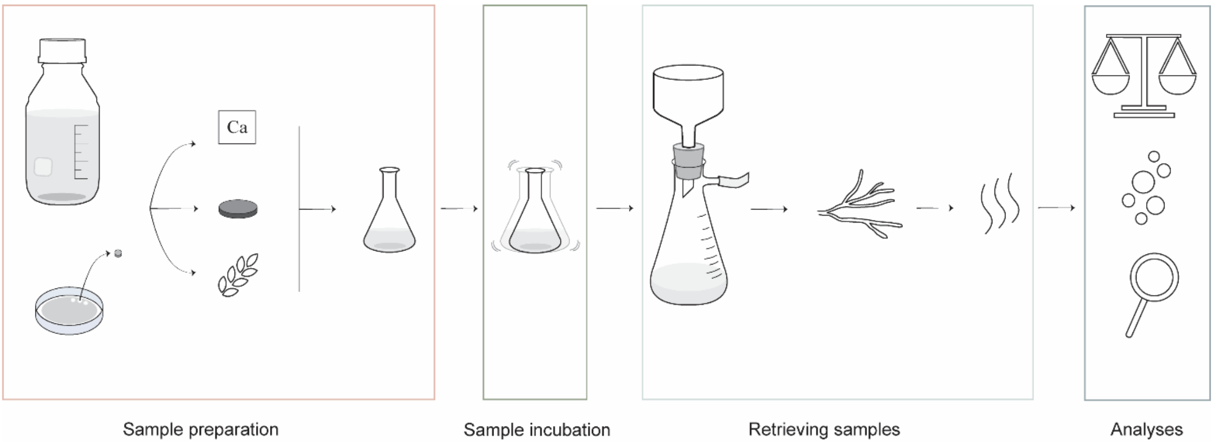

Supporting Figure S1. Conceptual overview of the experimental methodology in this study. The focus was placed on three different aspects: the calcium source and its concentration, the addition of cement and the type of nutrient. Based on these parameters, media were prepared, samples were inoculated and incubated in shaking conditions. With a vacuum pump, the biomass was retrieved. After killing and drying the biomass, different analyses were performed: quantification of the dry weight, quantification of the $CaCO_3$ precipitation with HCl dissolution and microscopic and chemical characterization of the samples.

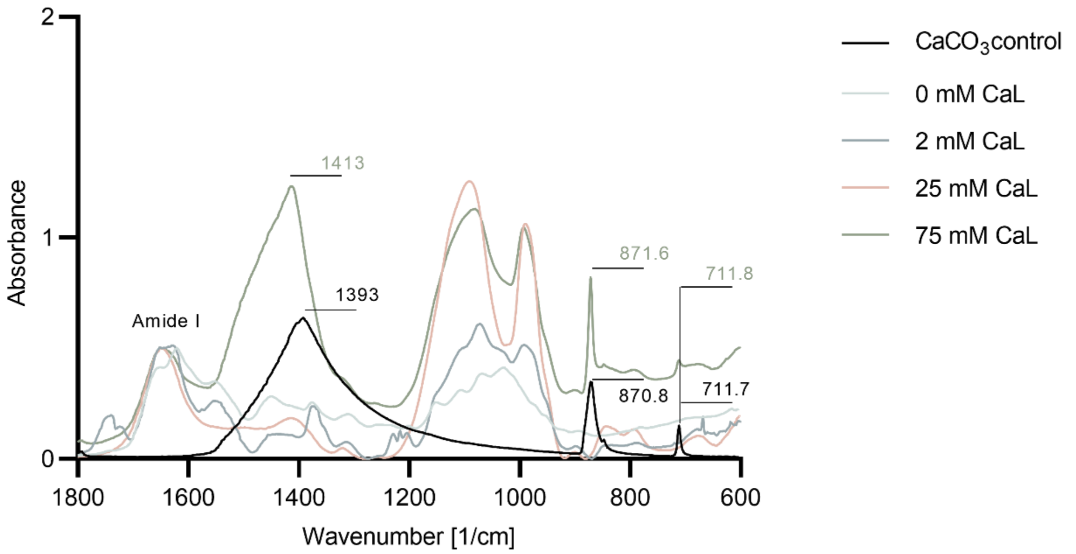

Supporting Figure S2. **Chemical analysis of $CaCO_3$ precipitation on fungal hyphae with FTIR.** Absorbance spectra are shown for samples from cultures cultivated with CaL**.** The wavelengths of the relevant peaks are indicated, as well as the Amide I region.

Supporting Table S1. Biomass and $CaCO_3$ formation upon growing *T. reesei* in presence of exogenous calcium sources. The mean values are given per calcium concentration and source.

	End pH value		$CaCO_3$ [mg]		Biomass [mg]	
	**$CaCl_2$**	**CaL**	**$CaCl_2$**	**CaL**	**$CaCl_2$**	**CaL**
**0 mM**	9.2	9.5	6.1	0.7	5.6	10.5
	(SD = 0.023)	(SD = 0.067)	(SD = 1.8)	(SD = 0.4)	(SD = 1.85)	(SD = 0.21)
**2 mM**	9.3	9.4	13.0	6.6	5.7	10.9
	(SD = 0.046)	(SD = 0.125)	(SD = 0.5)	(SD = 1.0)	(SD = 0.50)	(SD = 0.07)
**25 mM**	8.8	9.1	33.0	23.3	6.1	14.4
	(SD = 0.111)	(SD = 0.065)	(SD = 6.2)	(SD = 5.2)	(SD = 3.39)	(SD = 1.31)
**75 mM**	8.4	7.6	30.4	28.1	1.8	5.4
	(SD = 0.045)	(SD = 0.071)	(SD = 2.6)	(SD = 6.5)	(SD = 0.78)	(SD = 1.87)

Supporting Table S2. Biomass and $CaCO_3$ quantification upon growing *T. reesei* in presence of different media. The mean values are given per calcium concentration and source.

	End pH value		$CaCO_3$ [mg]		Biomass [mg]	
	**NB**	**B**	**NB**	**B**	**NB**	**B**
**CSL**	9.1	9.3	23.3	21.7	15.7	8.8
	(SD = 0.065)	(SD = 0.045)	(SD = 5.2)	(SD = 0.7)	(SD = 1.1)	(SD = 0.6)
**ME**	8.4	9.4	27.6	38.5	8.8	10.4
	(SD = 0.080)	(SD = 0.058)	(SD = 3.4)	(SD = 1.6)	(SD = 0.4)	(SD = 0.5)
**PD**	9.4	9.7	44.4	38.6	32.4	48.1
	(SD = 0.04)	(SD = 0.041)	(SD = 2.4)	(SD = 4.3)	(SD = 3.64)	(SD = 2.0)

Supporting Table S3. Overview of FTIR absorbance peaks that correspond to $CaCO_3$ precipitation in the experiments of samples from cultures grown in presence of different exogenous calcium sources. The peaks are compared to a control sample, consisting only of $CaCO_3$.

Sample	Peak 1	Peak 2	Peak 3
**$CaCO_3$**	**1393**	**870.8**	**711.7**
	$CaCl_2$		
0 mM	1413	/	/
2 mM	1416	/	/
**25 mM**	**1413**	**871.8**	**712.6**
**75 mM**	**1414**	**871.6**	**712.1**
	CaL		
0 mM	1376	/	/
2 mM	1375	/	/
25 mM	1416	/	/
**75 mM**	**1413**	**871.6**	**711.8**

Supporting Table S4. Overview of FTIR absorbance peaks that correspond to $CaCO_3$ precipitation in the experiments of samples from cultures grown in different media, in the presence of a pH buffer. The peaks are compared to a control sample, consisting only of $CaCO_3$.

Sample	Peak 1	Peak 2	Peak 3
**$CaCO_3$**	**1393**	**870.8**	**711.7**
CSL B	1407	/	/
ME B	1405	871.2	711.9
PD B	1408	871.6	711.5
